# The meso-fracturing mechanism of marble under unloading confining pressure paths and constant axial stress

**DOI:** 10.1038/s41598-021-97359-4

**Published:** 2021-09-08

**Authors:** Linna Sun, Liming Zhang, Yu Cong, Yaduo Song, Keqiang He

**Affiliations:** 1 School of Civil Engineering, Qingdao University of Technology, Qingdao, 266033 China; 2 Cooperative Innovation Center of Engineering Construction and Safety in Shandong Blue Economic Zone, Qingdao University of Technology, Qingdao, 266033 China; 3 School of Science, Qingdao University of Technology, Qingdao, 266033 China

**Keywords:** Natural hazards, Civil engineering

## Abstract

Failure tests on marble during unloading confining-pressure under constant axial stress and simulations with the particle flow code were performed. The influence mechanism of the unloading rate of the confining pressure, initial unloading stress, and confining pressure on the failure characteristics of, and crack propagation in, marble was studied. By using the trial-and-error method, the conversion relationship between the unloading rates of confining pressures in laboratory tests and numerical simulations was ascertained. Micro-cracks formed in the unloading process of confining pressure are dominated by tension cracks, accompanied by shear cracks. The propagation of shear cracks lags that of tension cracks. As the confining pressure is increased, more cracks occur upon failure of the samples. The proportion of shear cracks increases while that of tension cracks decreases. The failure mode of samples undergoes a transition from shear-dominated failure to conjugated shear failure.

## Introduction

The excavation, in underground engineering works, is a process of stress and strain release in a certain direction. Excavation unloading may induce a series of disasters in surrounding rock, such as a rockburst, caving, or spalling^1–6^. The failure characteristics of brittle rocks under an unloading path differ substantially from those under a loading path^6,7^. Brzovic found that the confining pressure first increases slightly and then gradually decreases during the excavation of a rock mass, so measuring mechanical parameters of rocks under an unloading path matches practical engineering scenarios^8^. Many researchers have found that rock brittle failure is more violent under unloading compared with that under loading^1–7^.

Previous research demonstrates that the stress state of rocks, unloading rate of confining pressures, and initial unloading stress all can influence the final failure modes of a rock mass^9,10^. By conducting rock failure tests at different unloading rates, Zhao et al. pointed out that the intensity of rock failure is related to the unloading rate^1^. Qiu et al. found that difference in failure characteristics of rocks under different stress unloading paths arises from changes in axial stress, so the unloading path under constant axial stress can better reflect the influence of confining-pressure unloading on the failure mechanism^2^. Experiments indicate that the behaviour of pre-cracked granite is more subject to changes in confining pressure at a low unloading rate of the confining pressure than that at a rapid unloading rate. The volumetric strain of rocks under unloading is larger than that when loading^9–13^.

To observe the development of cracks in rocks during unloading, new image recognition techniques have been used. A petrographic image analysis combined with qualitative microscopy can provide more reliable results for studying rock failure mechanics^14^. The micro-difference in the granite micro-cracks has been identified using a scanning electron microscope (SEM) under stress unloading paths^15^. By analysing computerised tomography (CT) images during unloading of rocks, Ren studied the evolution of damage during unloading ^16^. Huang et al. analysed the meso- and micro-morphological characteristics of failure planes of marble during unloading using three-dimensional laser scanning and the SEM ^17^. By means of nuclear magnetic resonance (NMR), Li et al. investigated the evolution of damage to rocks during unloading^18^.

While research into the micro-fracturing of rocks during unloading deepens our understanding of damage mechanisms incurred during unloading, it is not practical to conduct numerous repeated tests due to the cost of such testing schemes. To this end, numerical methods are used to study the failure mechanism of rocks during unloading ^19,20^. A simple finite element modelling approach was proposed for evaluating seismic energy release rate and strain energy storage rate. A new concept of critical strain energy is introduced to precinct the strainburst potential in mine^21^. A hybrid discrete-finite element method was adopted to analyze the effect of compressive strength of pillar, rock post-peak behavior, roof stiffness, and pillar and roof rock densities on the intensity of the strain burst^22^. Based on the numerical discontinuous deformation analysis method, Hatzor analyzed the total kinetic energy released and validate simulation results according to the monitored seismic energy emissions detected at Jinping II hydroelectric project^23^.

Particle flow code (PFC) is a discrete element method developed by Cundall and has been widely used in analysis of micro-fracturing of rock materials^24,25^. Compared with other software, PFC can better study the basic characteristics of rock-like materials, especially the fracture and crack development of rock-like media. Bahaaddini studied the mechanical properties of jointed rocks with different geometric parameters^26,27^. Huang used PFC to simulate the fracturing characteristics of rocks in the tensile-shear test^28^. Cai studied the mechanical behaviours of fractured rock masses under biaxial states of stress by combining finite element and discrete element methods^29^.

The results of PFC simulation conducted by different scholars are different^29^. With the increase of confining pressure, some scholars believe that the failure form is splitting transition to shear, while others find that shear transition to conjugate shear ^24–30^. The influence mechanism of unloading speed and initial position of unloading confining pressure on crack growth is not clear.

In-depth research into the failure mechanism of rock mass during unloading is the premise of accurately predicting the safety factor of surrounding rock masses. Considering that the macroscopic analysis fails to explain fully the failure mechanism of a rock mass during unloading, it was deemed necessary to study the mesoscopic failure mechanism of rocks during unloading. By conducting failure tests on marble during unloading confining pressures under constant axial stress, the crack evolution process inside the rock was reproduced in combination with the use of PFC. In this way, the influences of the unloading rate of confining pressures, initial unloading stress, and confining pressure on propagation of cracks in the rock were explored.

## Results of confining-pressure unloading under constant axial stress

### Test conditions

The tests were conducted on an MTS815 testing machine. The marble samples were machined into standard cylinders with a diameter of 50 mm and height of 100 mm, and the machining precision met test requirements^31^. The axial strain is measured using axial strain gauges and the circumferential strain is measured by way of circumferential chain-type strain gauges.

The marble used in the experiments had a fine-grained structure with a homogeneous texture. It is light red and contains mostly calcite and dolomite. The density and longitudinal wave velocity of the marble are 2.71 g/cm^3^ and 4100 m/s, respectively.

### Unloading paths

**Figure 1:**
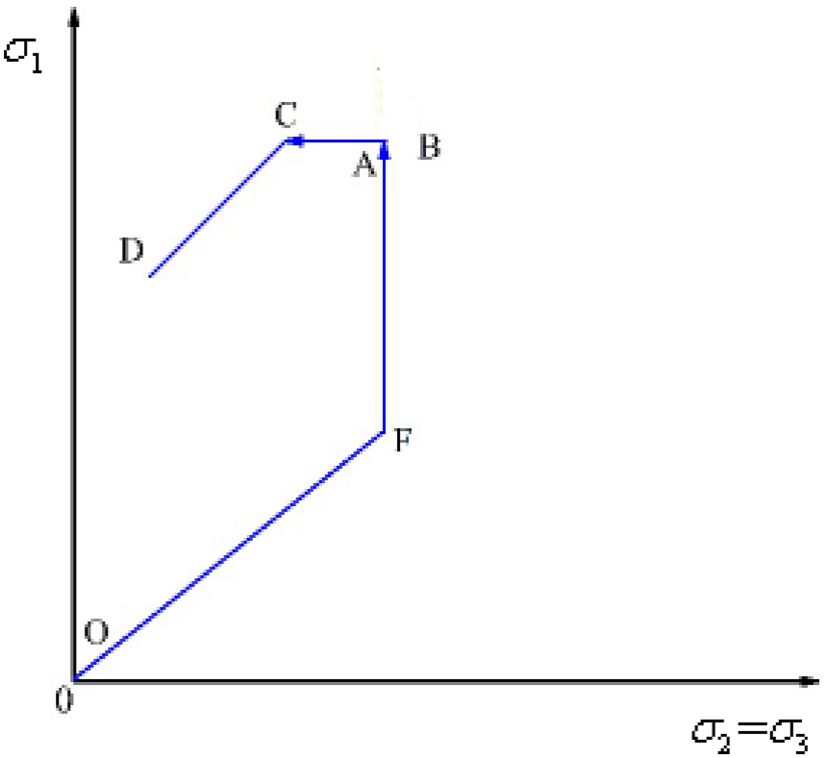
Stress paths in confining-pressure unloading tests under constant axial stress.

The paths in the confining-pressure unloading tests under constant axial stress are shown in Fig. 1. The test was performed according to the following steps.

A. An axial force of 2 kN was applied to the samples and then the confining pressure $\sigma_{{2}} = \sigma_{{3}}$ was applied until reaching the pre-set value (20 MPa).
B. Keeping the confining pressure $\sigma_{{2}} = \sigma_{{3}}$ unchanged, the axial stress $\sigma_{{1}}$ was increased to 80% of the peak strength at a rate of 0.002 mm/s under axial displacement control.
C. Keeping the axial stress unchanged, the confining pressure was reduced at different rates(0.2 MPa/s, 0.4 MPa/s, 0.6 MPa/s, 0.8 MPa/s) until the samples were completely damaged.

### Stress–strain curves

**Figure 2:**
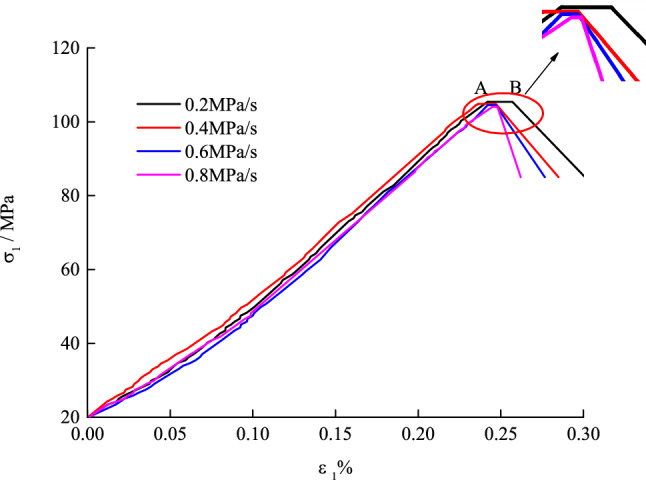
Stress–strain curves in the failure process of marble at different unloading rates.

Figure 2 illustrates the stress–strain curves in the failure process of the marble at different unloading rates of the confining pressure. After reaching the unloading point (80% of the peak strength), the lower the unloading rate, the longer the section with unchanged axial stress (from the unloading point A to point B) and the slower the rate of descent of the post-peak curve.

### Failure modes

**Figure 3:**
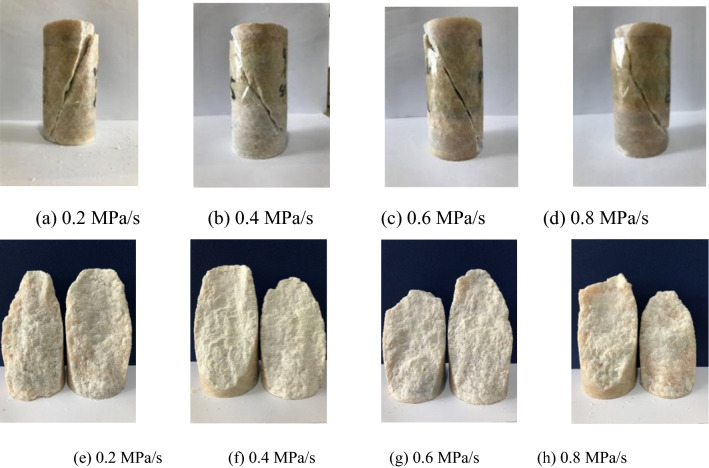
Failure modes and morphologies of failure planes of the marble during unloading of confining pressure at different rates.

Figure 3 shows the marble samples damaged at different unloading rates of the confining pressure. All samples are subjected to shear failure, with the failure angle of 62°–68°. The failure planes are uneven and rough.

## The PFC model for failure of marble during confining-pressure unloading

### Establishment of the mesoscopic model

**Figure 4:**
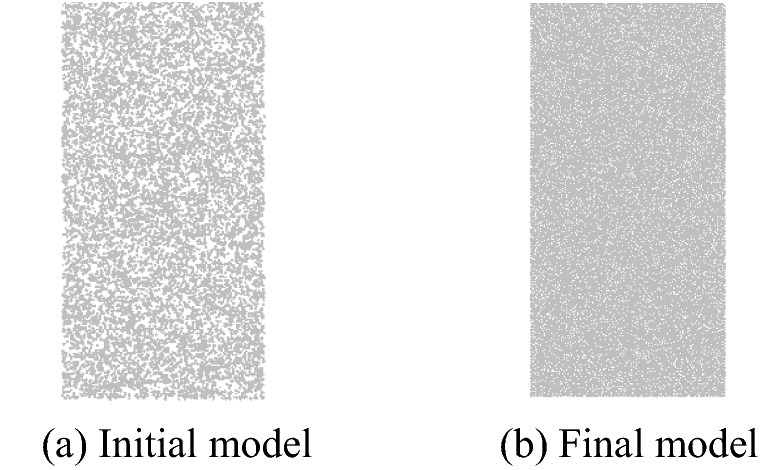
The PFC numerical model.

The parallel-bond model of the PFC was used to simulate the failure process of the marble during confining-pressure unloading^24,25^. The model was established and tested using the built-in language FISH of the PFC2D. The model measured 50 mm × 100 mm, containing 10,039 particles (Fig. 4). Setting the maximum and minimum radius of the particles, a specified number of particles within the size range of the initial model were generated. And under the control of the servo-motor, the final model was obtained by applying uniform pressure to the sample, the stress inside the model reached the equilibrium state and particles were uniformly distributed.

Four side walls were established, on which the confining pressure was applied to the pre-set value under servo-motor control. The axial load was developed through the face-to-face motion of the upper and lower surfaces, while the confining pressure was unloaded through reverse motion of the side walls. Different speeds corresponded to different unloading rates of the confining pressure.

The numerical simulation test was performed according to the following steps.

A. Four "walls" were generated, and using servo control to apply confining pressure to the four walls to a the pre-set value (20 MPa).
B. Keeping the confining pressure unchanged, through the top and bottom “walls” with constant velocity moves in opposite directions to simulate axial loading.
C. When the axial stress was loaded to 80% of the normal triaxial peak stress, using servo control to keep the axial pressure constant, and releasing the confining pressure at different rates (0.2 MPa/s, 0.4 MPa/s, 0.6 MPa/s, 0.8 MPa/s, 2.0 MPa/s, 4.0 MPa/s, 6.0 MPa/s, 8.0 MPa/s) until the specimen was broken.

### Determination of mesoscopic parameters

The mesoscopic parameters of the parallel-bond model include the bonding modulus $E_{{\text{c}}}$, parallel-bond modulus $\overline{E}_{{\text{c}}}$, stiffness ratio $K_{n} /K_{s}$ of particles, parallel-bond stiffness ratio $\overline{K}_{n} /\overline{K}_{s}$, parallel-bond radius multiplier, parallel-bond tensile strength strength $\overline{\sigma }_{c}$, parallel-bond cohesion strength $\overline{\tau }_{c}$, and friction coefficient $\mu$. To improve the accuracy of the simulation, the corresponding relationship between the mesoscopic and macroscopic parameters should be ascertained. In the present research, the trial-and-error method was used to determine parameters.

The trial-and-error method requires an analysis of the influences of mesoscopic parameters of a single parallel-bond model on the macroscopic mechanical properties, to build the qualitative relationship between macroscopic and mesoscopic parameters. Apart from this, it is necessary to study interactions of various parameters to establish relationships of parameters through fitting using multivariate functions based on results of numerical experiments. If values of the peak load, deformation parameters, and shear strength obtained in laboratory experiments are similar to those in numerical simulation, and the stress–strain evolution shows a similar trend in the two methods with consistent failure modes among samples, the group of mesoscopic parameters are deemed representative of the mechanical properties of rocks and are then used in subsequent numerical simulation. This group of parameters is considered optimal. Previous studies^28,32^ have provided the specific process for determining these parameters.

At first, a variable was adjusted while keeping other mesoscopic parameters unchanged. After determining the influence of the parameter on the macroscopic parameters, another mesoscopic variable was adjusted. The mesoscopic parameters determined using the trial-and-error method are listed in Table 1.

**Table 1:** Mesoscopic parameters of the marble.

Particles	Friction coefficient	Minimum particle size (mm)	Radius ratio	Particle density (g/cm^3^)	Contact modulus of particles (GPa)	Stiffness ratio of particles
	0.50	0.30	1.66	2670	20.0	2.00

Bonding	Parallel-bond radius multiplier	Parallel-bond modulus (GPa)	Parallel-bond stiffness ratio	Parallel-bond tensile strength (MPa)	Parallel-bond cohesion strength (MPa)	Internal friction angle (°)
	1	24.0	2.00	42	21	41

**Figure 5:**
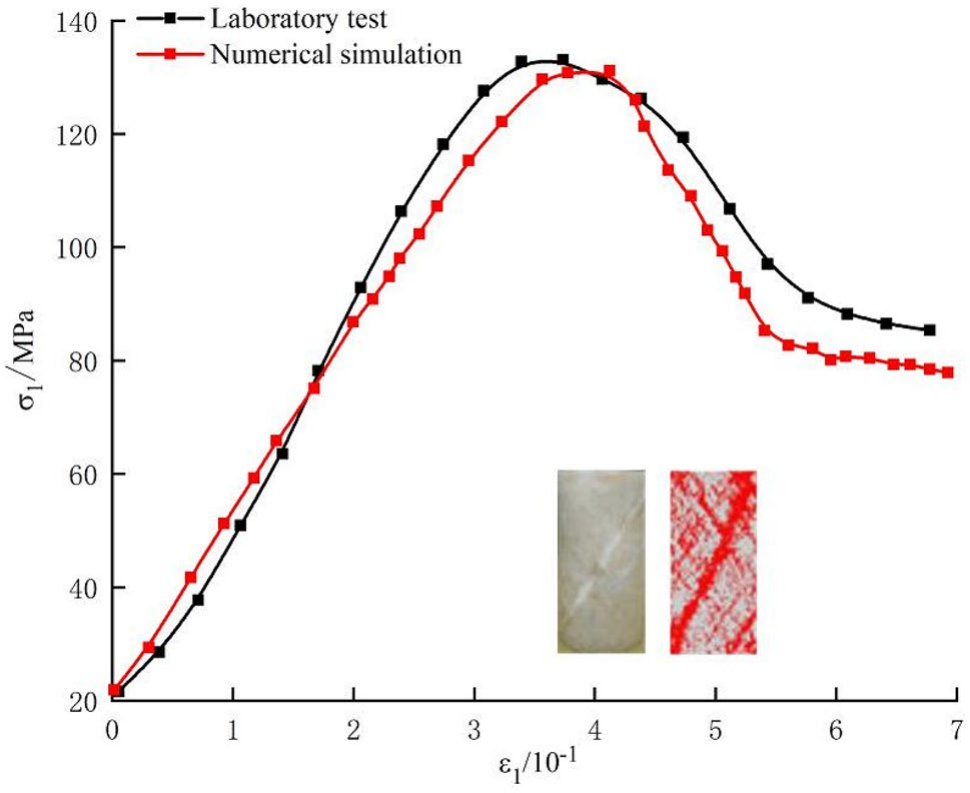
Comparison with the stress–strain curve and failure modes in the conventional triaxial test.

Figure 5 shows the stress–strain curves and failure modes of the marble during the numerical simulations and laboratory tests under a confining pressure of 20 MPa. Except for failure in reproducing the compaction characteristics of the samples, the PFC simulations yield results in good agreement with the laboratory test in terms of the overall shape of the curves, peak strength, peak strain, and failure modes. The results indicate that the parameters used can simulate the failure mode of the marble.

## Analysis of microscopic crack failure process of marble during unloading confining pressure

### Influence of unloading rate

#### Characteristics of stress–strain curves

**Figure 6:**
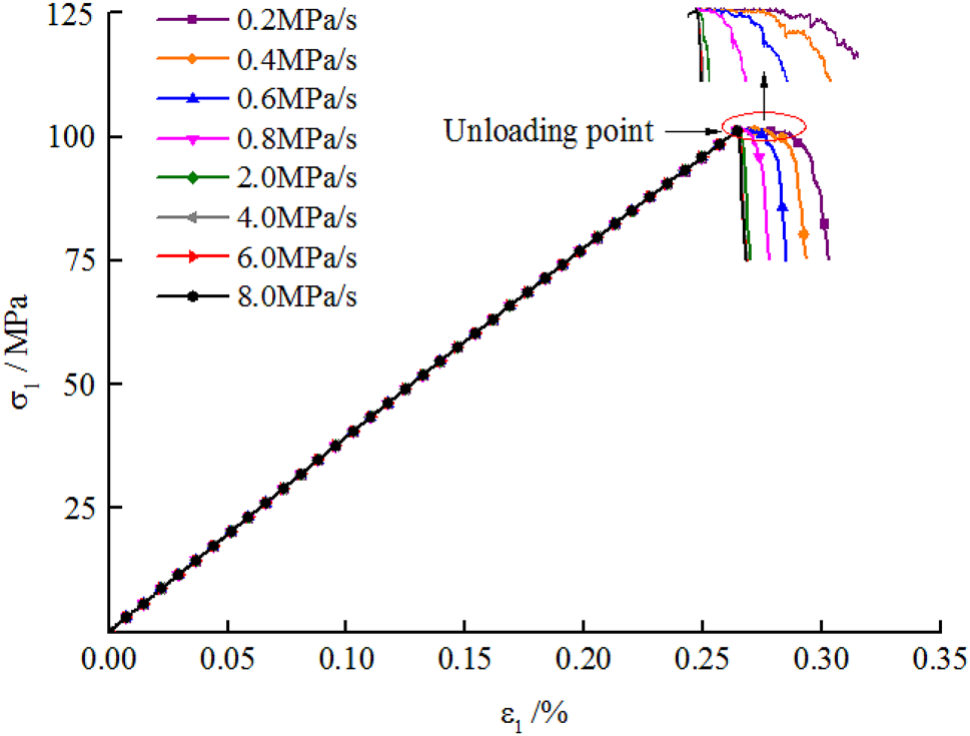
Numerically simulated stress–strain curves during failure of marble at different unloading rates.

The numerically simulated stress–strain curves for failure of the marble during unloading confining-pressure under a confining pressure of 20 MPa are shown in Fig. 6, with the overall shape of the curves remaining consistent with test data (Fig. 2). The axial stress was kept unchanged after confining-pressure unloading began. The stress–strain curves exhibited a plateau, and the stress decreased abruptly after failure. It can be seen from the locally enlarged inset that the lower the unloading rate of the confining pressure, the longer the time during which the stress–strain curves remained unchanged and the lower the post-peak rate of decrease of stress. The stress–strain curves were similar when the unloading rates were between 4 and 8 MPa/s and the shape of the curve did not change with the unloading rate.

#### Number of cracks

Failure defined in the PFC only contains tensile failure and shear failure^24,25^. It is possible to determine that what appear to be tensile or shear fractures on a large scale are produced through a combination of tensile and shear mechanisms on a smaller scale. In the PFC, the internal stress of the model is calculated by iteration. Tensile failure at the contacts between particles occurs when the maximum tensile stress exceeds the parallel-bond tensile strength; shear failure occurs if the maximum shear stress exceeds the parallel-bond shear strength, therefore, in the present research, micro-cracks are only divided into two types: tension cracks and shear cracks.

**Figure 7:**
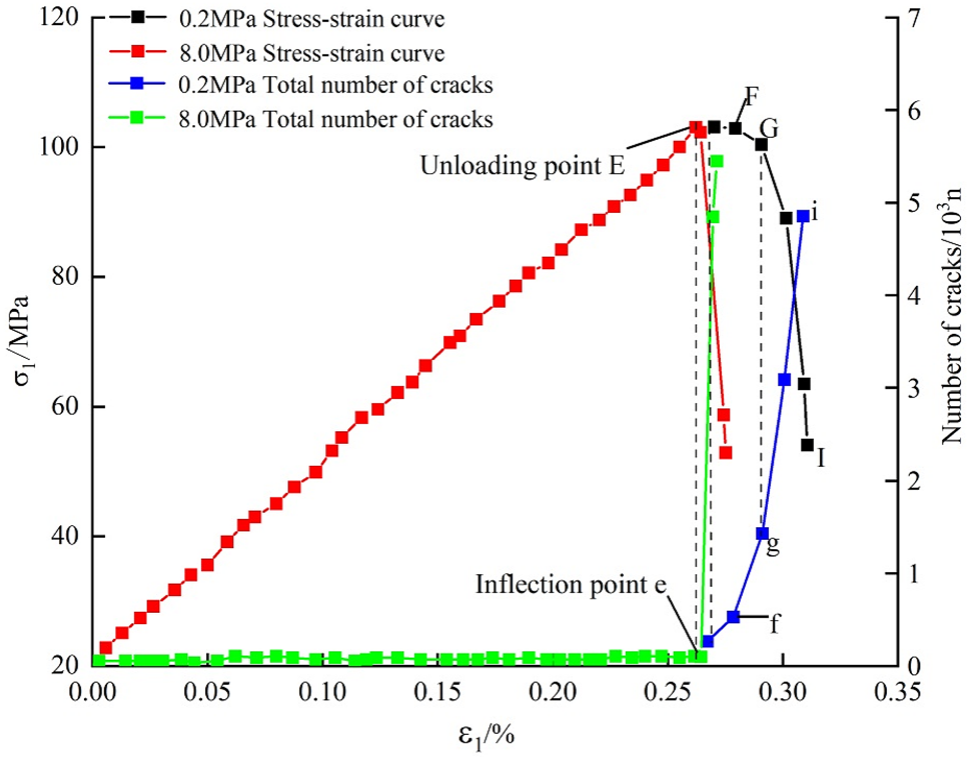
The stress-crack number-strain curves at different unloading rates of the confining pressure.

Figure 7 shows the stress-crack number-strain curves during failure of the marble at different unloading rates under a confining pressure of 20 MPa. In the initial stage of loading, few cracks appear in the rock, and the crack number-strain curves increase slowly (section OE). After the confining-pressure unloading begins, an inflection point (E) appears on the curves. At an unloading rate of 8.0 MPa/s, the plateau of the curves under constant axial stress is short and then the stress drops (section FH), when the total number of cracks is 146 (at point F). At a low unloading rate (0.2 MPa/s), there are 1010 cracks in the plateau of the curves under constant axial stress (at point G), which is 6.9 times the number at a high unloading rate. As the unloading finishes, the increase in the total number of cracks at a high unloading rate is shown as a vertical curve in the figure, with the rate of growth of the number of cracks being much greater than that at a low unloading rate. The rock samples are deformed slightly at a high unloading rate, and the samples exhibit abruptly declining strength in a short time and are violently damaged.

**Figure 8:**
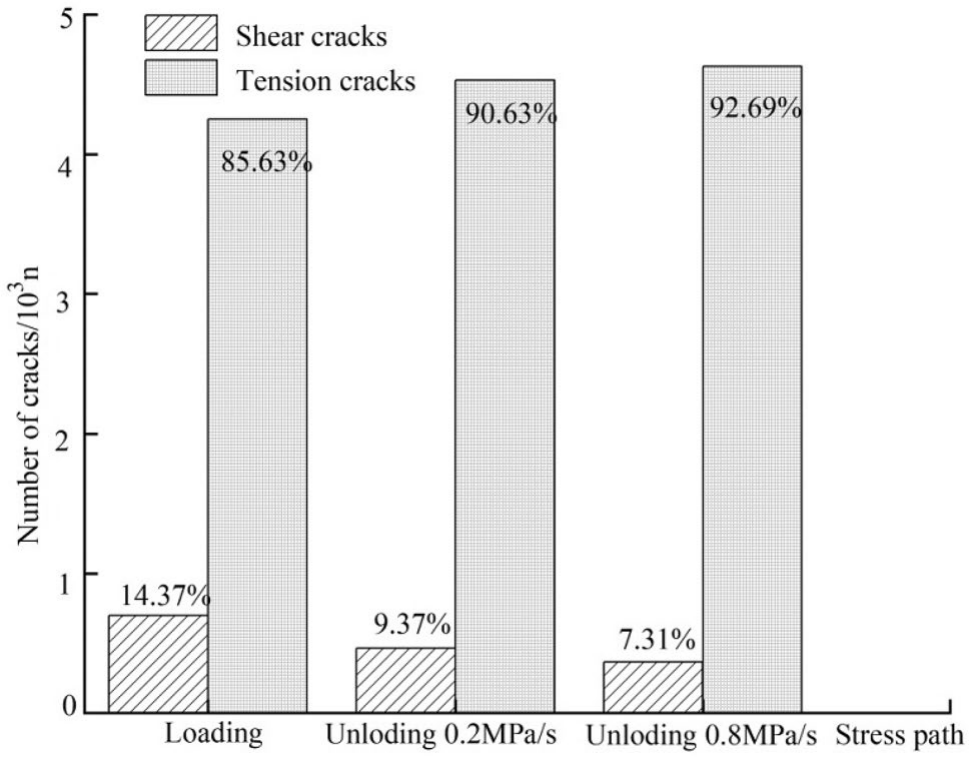
Bar graph of the number of cracks at different unloading rates of confining pressure.

Figure 8 is a bar graph showing the number of cracks during failure of the marble at different rates of unloading under a confining pressure of 20 MPa. During conventional triaxial loading to failure, the shear cracks and tension cracks separately account for 14.37% and 85.63% of the total. As the unloading rate is increased, the proportion of tension cracks grows from 90.63 to 92.69%, while that of shear cracks decreases from 9.37 to 7.31%, suggesting that the high unloading rate is more likely to generate tension cracks.

#### Failure modes

**Figure 9:**
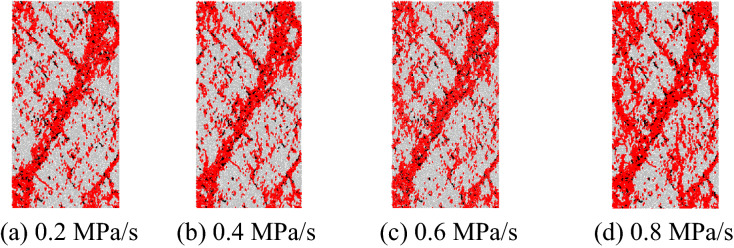
Distribution of mesoscopic cracks during failure of the marble at different unloading rates.

Figure 9 shows the distribution of mesoscopic cracks in the marble during failure at different unloading rates of the confining pressure, where red and black represent tension cracks and shear cracks, respectively. The rock is dominated by shear failure and the dominant shear planes are surrounded by secondary cracks. There is a small number of secondary cracks at a low unloading rate of 0.2 MPa/s. As the unloading rate is increased, the secondary cracks become more numerous and coalesce to form secondary failure planes. The higher the unloading rate, the larger the amount of secondary failure planes. This is because the effect of confining pressure on the sample is gradually diminished due to confining-pressure unloading. The faster the confining-pressure unloading, the more rapid the decrease of confining pressure and the faster the transverse motion of particles. As a result, the tendency of cracks to propagate along the dominant shear planes reduces and secondary cracks are more apt to extend transversely.

### Influence of the initial unloading stress

#### Number of cracks

**Figure 10:**
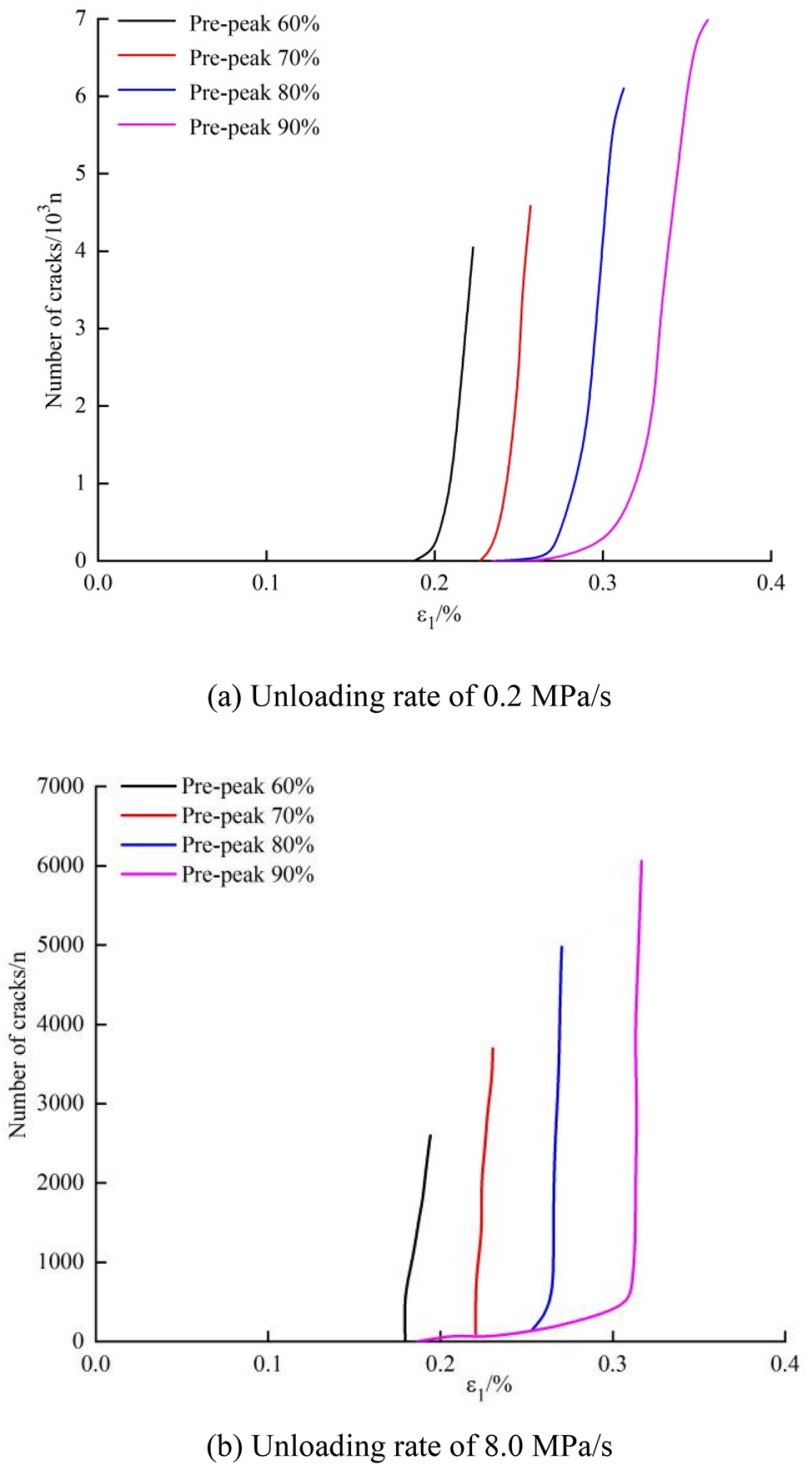
Crack number-strain curves of the marble during failure at different initial unloading stresses of the confining pressure.

Figure 10 shows the crack number-strain curves of the marble during failure with different initial unloading stresses. The initial unloading stresses are 60%, 70%, 80%, and 90% of the peak strength pre-peak, and the unloading process is ended when the axial stress reaches 60% thereof post-peak. The stress–strain curves are similar, with an initial slow, then rapid increasing trend. The closer the initial unloading stress to the peak strength, the longer the slow increase process of the curve in the initial stage of deformation and the later the abrupt growth of the crack number in the late stage. The rate of growth of cracks at a high unloading rate is greater than that at a low unloading rate. This indicates that, at the same initial unloading stress, the high unloading rate exerts a greater effect on crack propagation.

#### Failure modes

**Figure 11:**
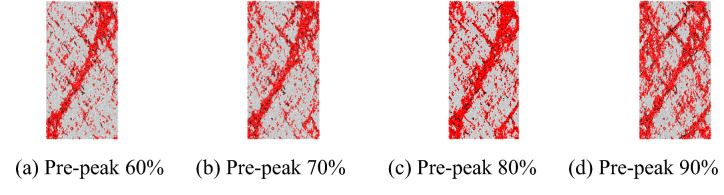
Failure modes of the marble at different initial unloading stresses.

The failure modes of the marble at different initial unloading stresses and an unloading rate of 0.2 MPa/s are shown in Fig. 11. The axial stress is kept unchanged as the confining pressure is varied between 60 and 90% of the peak strength pre-peak. The failure mode of the marble does not change when increasing the initial unloading stress of the confining pressure, while the number of cracks increases significantly.

### Influence of the confining pressure

#### Number of cracks

**Figure 12:**
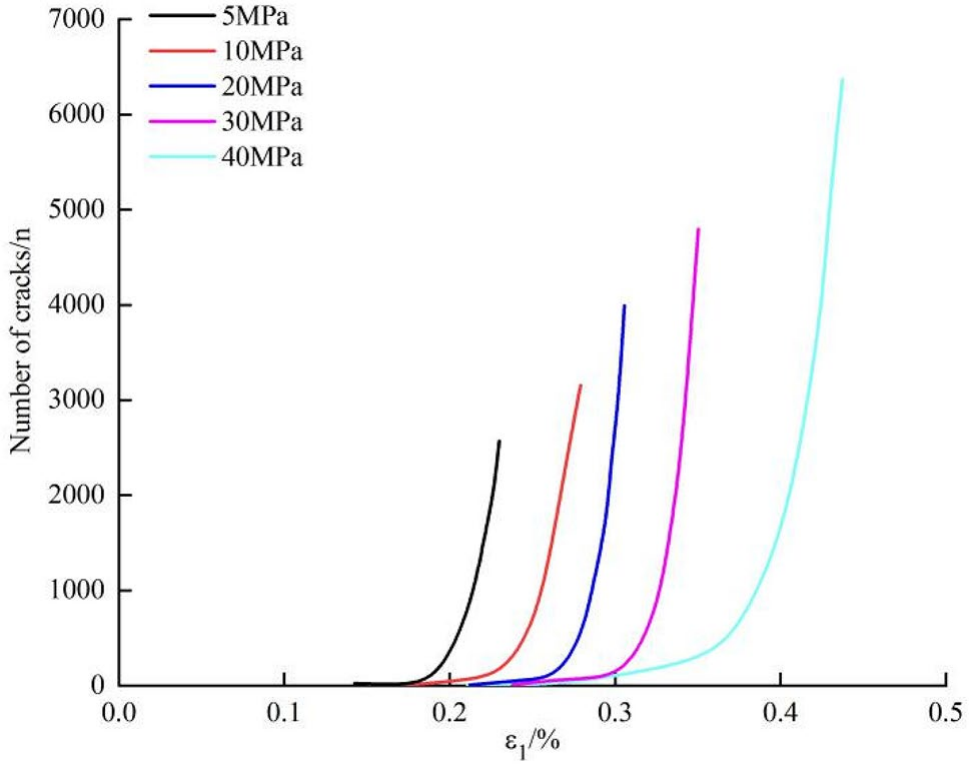
Crack number-strain curves during failure of the marble under different confining pressures.

The overall shape of the crack number-strain curves during failure of the marble under different confining pressures is similar (Fig. 12). As the confining pressure is increased from 5 to 40 MPa, the number of cracks grows slowly in the initial stage of deformation, while increasing abruptly in the later stages thereof. The larger the confining pressure, the longer the period of slow increase in deformation, indicating that a high confining pressure can inhibit crack propagation in the initial stages of testing.

**Figure 13:**
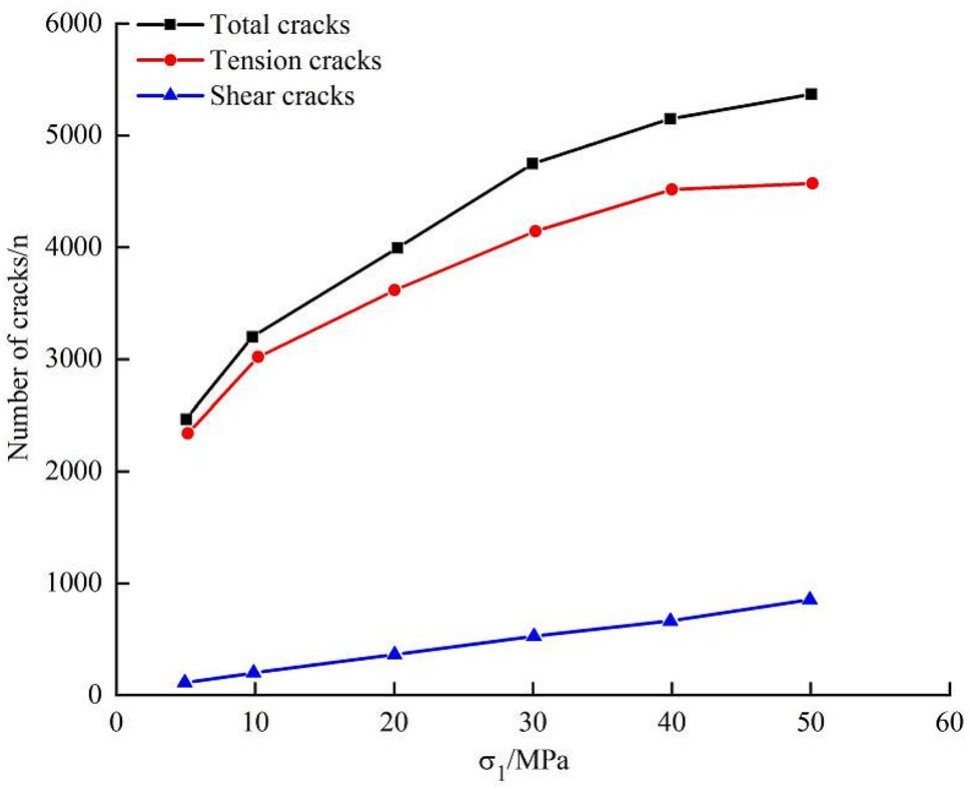
Crack number-confining pressure relationships during failure of the marble under confining-pressure unloading.

Figure 13 illustrates the crack number-confining pressure relationships during failure of the marble under confining-pressure unloading. As shown, the confining pressure, total number of cracks, number of tension cracks, and number of shear cracks all exhibit an increasing trend, while the rates of growth of the total number of cracks and the number of tension cracks decrease, and the rate of growth of the number of shear cracks remains unchanged. This suggests that, as the confining pressure is increased, the proportion of shear cracks among all cracks increases while that of the tension cracks falls. At a confining pressure of 5 MPa, there are 163 shear cracks, which account for 5.6% of the total, and 1747 tension cracks (94.4% of the total); while at a confining pressure of 50 MPa, there are 812 shear cracks and 4050 tension cracks, 16.7% and 83.3% of the total.

#### Failure modes

**Figure 14:**
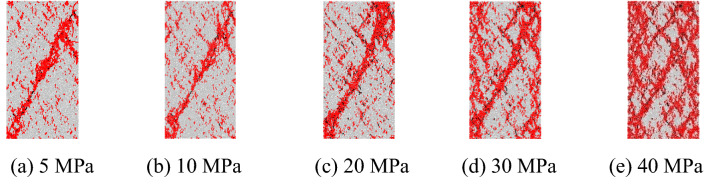
Mesoscopic failure modes of the marble under different confining pressures.

The failure modes of the marble under unloading from different confining pressures are displayed in Fig. 14. Shear-dominated failure is found at low confining pressures; as the confining pressure is increased, the amount of secondary failure planes increases around the dominant shear planes, showing a transition from shear-dominated failure to conjugated shear failure.

## Discussion

**Figure 15:**
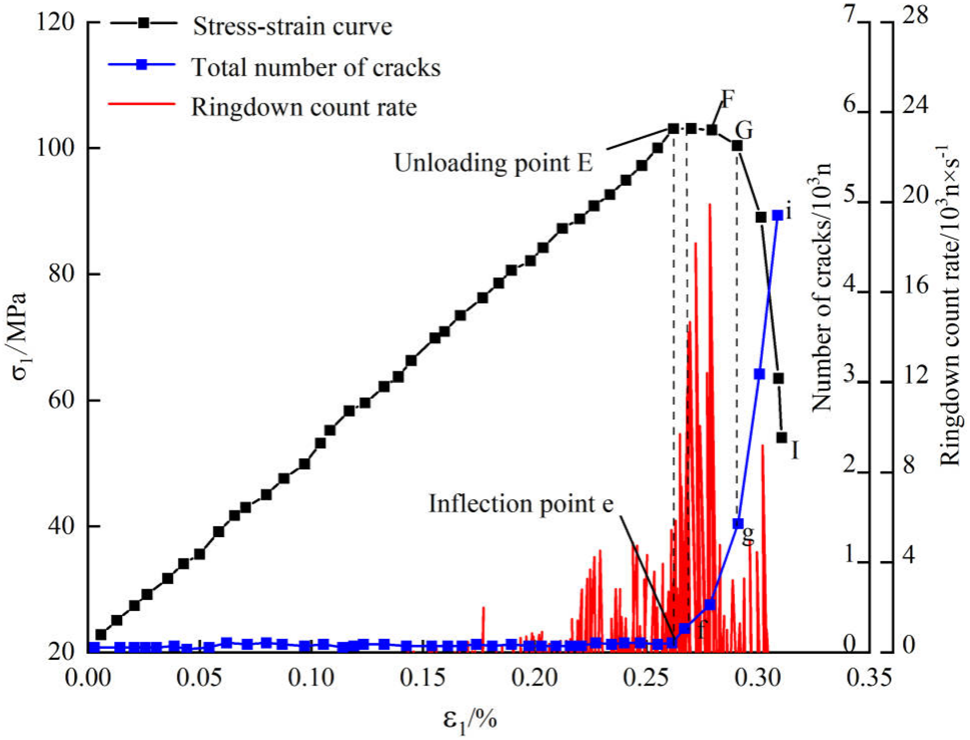
The relation between stress, crack number, ringdown count rate and axial strain at unloading rate 0.2 MPa/s.

AE21C acoustic emission testing equipment was used to monitor the acoustic emission information during the deformation of marble^33^. The ringdown count rate data of samples at failure at a confining pressure of 20 MPa and an unloading rate of the confining pressure of 0.2 MPa/s (Fig. 15) were analyzed. The ringdown count rate does not grow continuously but in an irregular manner. Each incidence of a large ringdown count rate is a result of the energy release from crack propagation and coalescence. The ringdown count rate is low before the elastic stage. The ringdown count rate experiences stepwise growth when the applied load is increased to the crack-initiation stress. In that case, the number of cracks begins to increase and the corresponding ringdown count rate also starts to increase in both quantity and amplitude. The confining pressure is unloaded from 80% of the peak strength, when the ringdown count rate grown unsteadily again, remaining at a high value. Under these conditions, the number of cracks also begins to increase in a non-linear manner. Unloading of the confining pressure results in the rapid increase of circumferential deformation and the decentralized micro-cracks continue to extend and coalesce to form macro-cracks, such that the sample is rapidly damaged. It is evident that the change in the ringdown count rate accords completely with that in the number of cracks, validating the correctness of the simulation results with PFC.

Micro-cracks formed in the unloading process of confining pressure are dominated by tension cracks, accompanied by shear cracks. The number of tension cracks is much greater than that of the shear cracks in the deformation process and the propagation of shear cracks lags that of tension cracks. As the unloading rate increases, the proportion of tension cracks increases and the secondary cracks are more likely to extend transversely, so tensile failure is more apt to occur. With the increase in the unloading rate, the number of cracks decreases sharply and influences of the unloading rate on the failure of samples are mainly embodied by changes in the rate of crack propagation.

As the confining pressure is increased, the slow increase in cracking is prolonged and more cracks occur upon failure of the samples. The proportion of shear cracks increases while that of tension cracks decreases. As a result, the failure mode of samples undergoes a transition from shear-dominated failure to conjugated shear failure. With increasing confining pressure, the unloading rate exerts more significant influences on the predicted failure mode.

**Figure 16:**
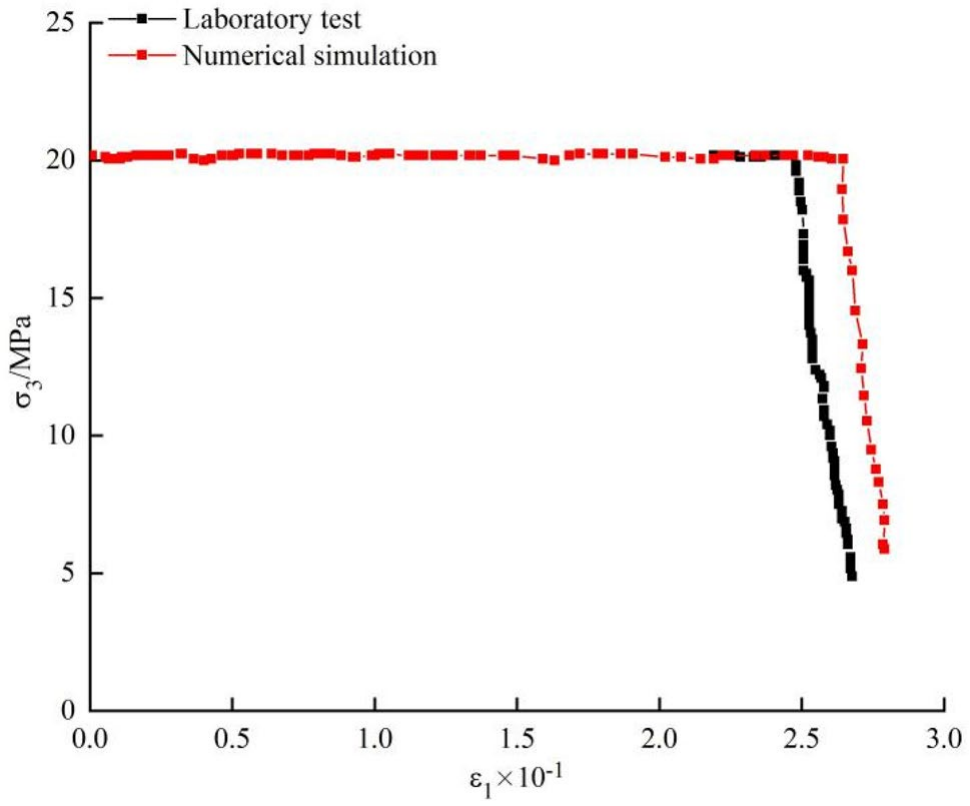
Confining pressure-axial strain curves in laboratory tests and PFC simulations.

In laboratory tests, the unloading rate of the confining pressure was measured in MPa/s, while that in PFC simulations is in MPa/step. Two opposite side walls can move in the opposite directions at a certain speed, so that they move outward to simulate the unloading process. If the walls move too slowly, they change direction before completing the unloading process. As a result, the confining pressure-axial strain curves grow in the opposite direction to that seen in conventional tests. If the two opposite side walls move too fast, it does not conform to the preset unloading rate in the laboratory test. Through trials, the measurement units of laboratory tests and PFC simulations are converted thus: under the same confining pressure, if the confining pressure-axial strain curves in laboratory tests and PFC simulations have consistent slopes during the unloading confining process and their unloading rates are deemed equal, as shown in Fig. 16. Finally, it is found that the measurement units of laboratory tests and PFC simulations are converted using 1 MPa/s as equivalent to 0.006 MPa/step.

## Conclusion

The failure process of the marble was reproduced through DEM. According to the confining pressure-axial strain relationship, the measurement units in the laboratory tests and PFC simulations are converted as 1 MPa/s = 0.006 MPa/step, which overcomes the difficulty in quantitative control of the unloading rate in PFC.

Micro-cracks formed in the unloading process of confining pressure are dominated by tension cracks, accompanied by shear cracks. The number of tension cracks is much greater than that of the shear cracks in the deformation process and the propagation of shear cracks lags that of tension cracks. As the unloading rate increases, tensile failure is more apt to occur.

As the confining pressure is increased, more cracks occur upon failure of the samples. The proportion of shear cracks increases while that of tension cracks decreases. The failure mode of samples undergoes a transition from shear-dominated failure to conjugated shear failure.
